# A Novel Carboxyl-Terminal Heptapeptide Initiates the Regulated Secretion of LH from Unique Sub-Domains of the ER

**DOI:** 10.1371/journal.pone.0065002

**Published:** 2013-05-29

**Authors:** Albina Jablonka-Shariff, Irving Boime

**Affiliations:** Departments of Developmental Biology and Obstetrics and Gynecology, Washington University School of Medicine, St. Louis, Missouri, United States of America; Institute of Molecular and Cell Biology, Singapore

## Abstract

The coordinated secretion of LH and FSH are critical for reproductive functions. After translocation into the endoplasmic reticulum (ER), their biosynthetic routes diverge at a determinative step prior to sorting in the regulated (LH) and constitutive (FSH) secretion pathways. Recently, we identified a C-terminal heptapeptide sequence, present only in the LHβ subunit, as a critical signal for entry of the LH dimer into the regulated pathway. We showed that an LHβ mutant lacking the heptapeptide (LHβΔT) assembled more efficiently with the α subunit than wild-type LHβ subunit, and this LHΔT dimer was secreted constitutively. Thus, an association exists between the presence of the C-terminal heptapeptide and sorting of the LH heterodimer to the regulated pathway. To study how this delayed LHβ subunit assembly is related to the trafficking of LH, we exploited the single subunit transfection model in rat somatotrope-derived GH_3_ cells with the use of immunofluorescence confocal microscopy. The LHβ subunit showed a distinct immunofluorescent localization as compared to the FSHβ subunit and LHβ mutants. The wild-type LHβ subunit exhibited a perinuclear staining corresponding to the ER/nuclear envelope region. In contrast, the wild-type FSHβ subunit and the mutants LHβΔT and LHβL119A displayed no detectable perinuclear staining; only peripheral ER puncta were observed. Also, no perinuclear fluorescence was detected in cells expressing the LH heterodimer. We propose that the C-terminal heptapeptide is responsible for delayed heterodimer assembly within an ER sub-domain of the nuclear envelope, as an early partitioning event necessary for the entrance of LH into the regulated secretory pathway, whereas FSHβ does not traverse the nuclear envelope region. These data suggest that, at least for LH, the molecular decision to enter the regulated secretory pathway is a pre-Golgi event controlled by the novel C-terminal heptapeptide.

## Introduction

The glycoprotein hormone family includes the pituitary LH, FSH and TSH and the placental hormone hCG. LH and FSH, essential for normal follicular development and ovulation, are synthesized in the same gonadotrope cell, but their secretion pathways differ. Following exit from the Golgi complex, LH is stored in dense core granules and is released in pulses via the regulated pathway in response to gonadotropin releasing hormone [1], [2]. In contrast, FSH is secreted primarily through the constitutive pathway and approximates its biosynthetic rate [3]–[5]. That secretion of LH and FSH overlaps at the pre-ovulatory surge of the estrous cycle [6], [7], raises the fundamental question as to how two structurally related gonadotropin hormones are released from the same cells through distinct secretory routes. Defining the early signals that govern the unique intracellular trafficking routes of LH and FSH and to understand the mechanistic link between their secretion and reproductive function has been a major goal of our laboratory [8]–[10] and others [11]–[15].

This entire gonadotropin quartet is comprised of heterodimers that share a common α subunit but differ in their hormone-specific β subunits. Thus, it was reasonable to conclude that the β subunit contains the trafficking cues responsible for diverting LH and FSH to their respective secretory pathways. In support of this, we reported that the C-terminal heptapeptide in the LHβ subunit, not found in the FSHβ subunit, is essential for the regulated release of the LH dimer [16]–[18]. The manner in which this peptide functions as a sorting signal, however, is not clear.

It is known that β/α subunit assembly occurs within the ER lumen [19], [20]. Earlier observations that might explain the mechanism of the LHβ heptapeptide demonstrated that unassembled pituitary β subunits do not efficiently exit the ER in the absence of the α subunit [9], [10], [21]. Although co-expression with the α subunit rescued the β subunits, there were major differences in the extent of assembly of the β/α subunit pairs. For example, whereas more than 80% of the FSH dimer was generated and subsequently secreted [21], less than 10% of the LH dimer was formed [8], [22]. The conclusion was that the LHβ heptapeptide accounted for this inefficient assembly. Taken together, these data imply a link between LHβ/α assembly and the sorting step for LH, both of which depend on the presence of the C-terminal heptapeptide. To address this hypothesis, we performed a series of morphological studies using the rat somatotrope-derived GH_3_ cell line, which contains both, regulated and constitutive secretion pathways. We used immunofluorescent confocal analysis of clones expressing single unassembled LHβ and FSHβ subunits, and their corresponding mutants. In support of this model, we demonstrate that the newly synthesized LHβ subunit localizes to the ER/nuclear envelope (NE) region, while the FSHβ subunit displays no detectable perinuclear staining, but only peripheral ER distribution. Taken together, the C-terminal heptapeptide is responsible for directing LH to the regulated secretory pathway via the ER/NE region, whereas the initiation of FSH trafficking involves a different locus of the ER. The implication of these novel data is that, at least for LH, the decision to enter the regulated pathway involves a pre-Golgi event prior to entering the trans-Golgi network as is traditionally believed.

## Results

**Figure 1:**
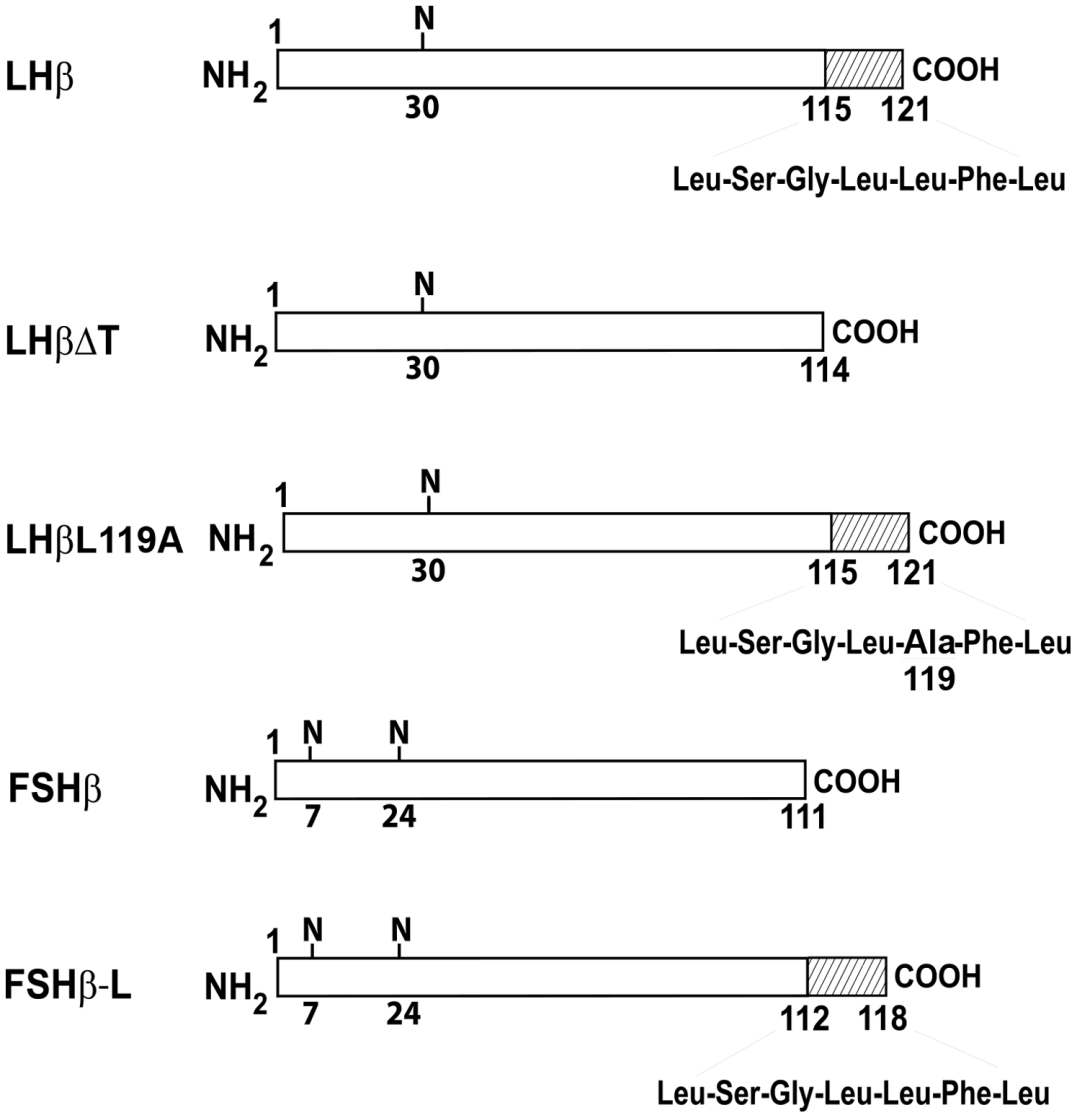
Schematic diagram of human gonadotropin subunits. The crosshatched area of the region 115–121 denotes the heptapeptide of the LHβ subunit. N, Asn-linked oligosaccharides.

**Figure 2:**
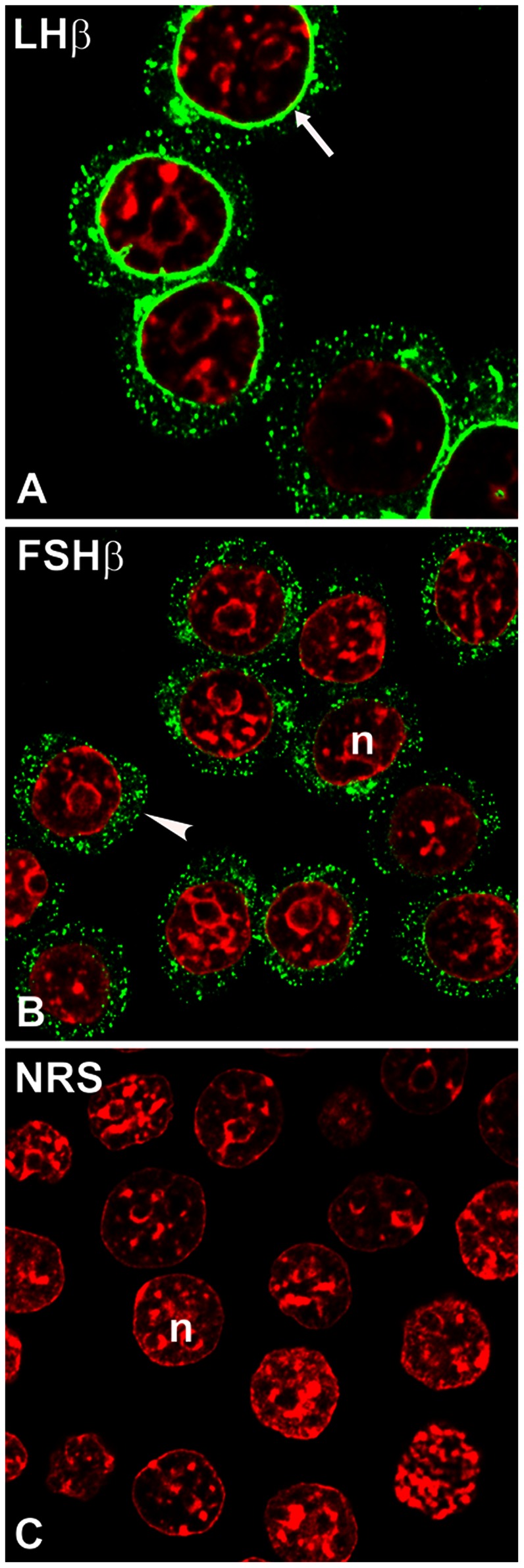
Subcellular localization of LHβ (A) and FSHβ (B) subunit in GH_3_ cells. The cells were immunostained with CGβ antiserum (*green*) and monoclonal antibody against FSHβ subunit (*green*). *Note* unique ER/perinuclear staining pattern for LHβ (A, *arrow*) vs. dispersed cytoplasmic puncta for FSHβ subunit (B, *arrowhead*). The n indicates the nucleus (*red*). The micrographs shown are representative of four to eight experiments and are at the X100 and X150 magnification. NRS (C), normal rabbit serum.

**Figure 3:**
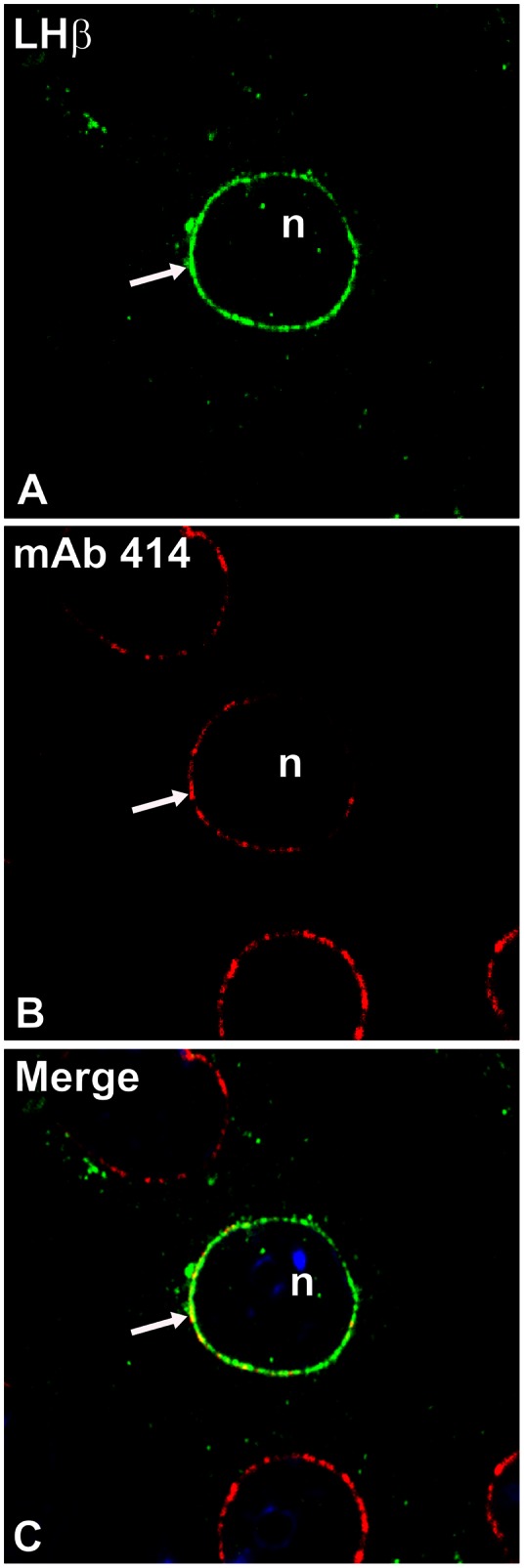
Co-localization of LHβ subunit with a nuclear envelope marker (A–C). GH_3_ cells expressing the LHβ subunit were immunostained with CGβ antiserum (A, *green*) and mAb 414 (B, *red*). The merged image (C) indicates co-staining of LHβ subunit with the nuclear pore complex proteins (*yellow, arrow*). Nuclei (n) were counterstained using TOPRO-iodide-3 (*blue* shown only in C). These images are representative of four independent experiments. X150.

Previous studies from our laboratory revealed that the C-terminal heptapeptide in the LHβ subunit functions as a sorting determinant for the regulated secretion of the LH heterodimer [16], [17]. Deletion of this heptapeptide from the LHβ subunit (LHβΔT, Fig. 1) led to a constitutively secreted LHΔT dimer [16]. To investigate the function of the heptapeptide in the sorting pathway, confocal immunofluorescence staining was performed in GH_3_ cells expressing single unassembled LHβ and FSHβ subunits and mutants. When comparing the LHβ and FSHβ staining patterns (Fig. 2) the most striking feature is the perinuclear localization of LHβ (70.1±3.3% of cells; >200 cells; Fig. 2A), whereas FSHβ displays only a pattern of dispersed cytoplasmic puncta (Fig. 2B). No detectable staining was seen when normal rabbit serum (NRS) was substituted for the LHβ immuno probe (Fig. 2C). To verify that the LHβ staining was confined to the NE region, we co-stained LHβ with a known marker of the NE [23], a monoclonal antibody against nuclear pore complex proteins designated mAb414 (Fig. 3). It is clear that this marker delineates the NE (Fig. 3B). Merged images confirmed that the LHβ subunit is localized in the NE region (Fig. 3C). It is unclear why the LHβ staining is not more uniform and exhibits a clustering at the NE. It may be related to an incomplete ER overlap at the NE since staining mAb 414 shows a relative uniform punctate staining of the NE. The numerous punctate images of the LHβ and FSHβ subunits do not correspond to the expected diffused network of ER. However that their staining is similar to the ER marker, calnexin (see below) shows that the subunits reside in the ER. Since less that 10% of the LHβ and FSHβ subunits are secreted, and the majority of the pool accumulates in the ER, not freely diffusible in the lumen, it is likely that these subunits are bound to a component in the ER and/or accumulate at ER exit sites. In any case the issue is that the distinctions in the biosynthetic pathways are initiated at an earlier stage.

**Figure 4:**
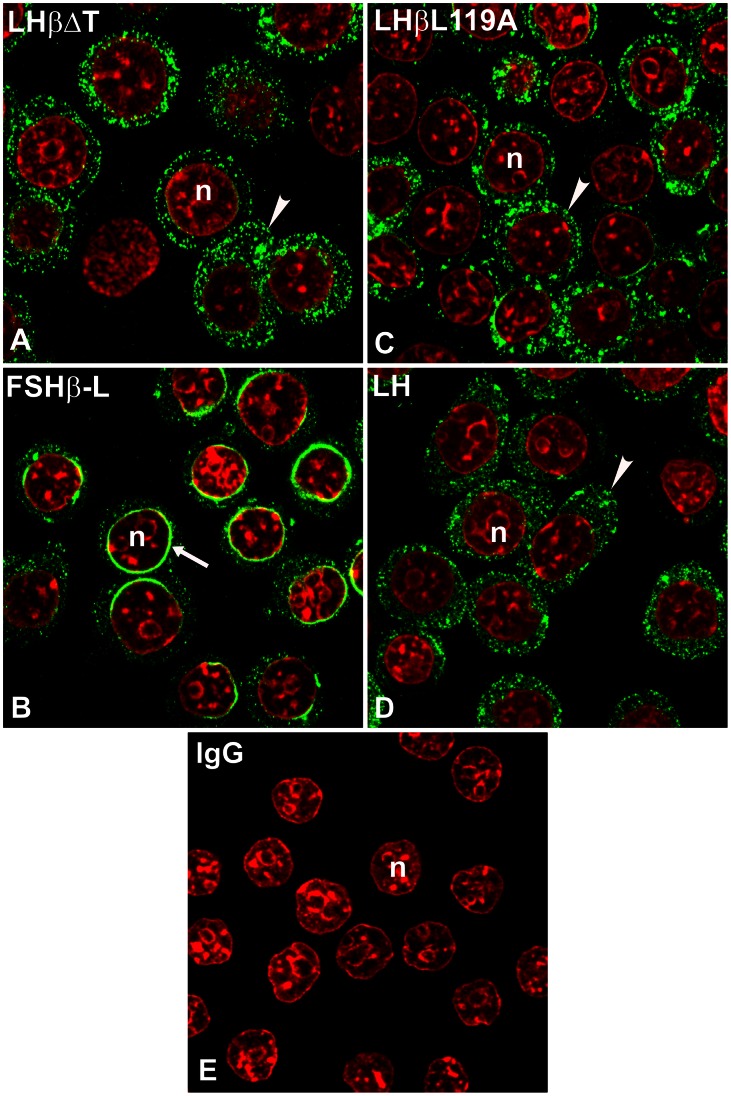
Subcellular localization of LHβΔT (A), FSHβ-L (B), LHβL119A (C) subunit and LH dimer (D) in GH_3_ cells. The cells were immunostained with CGβ antiserum (*green*) and a monoclonal antibody against FSHβ subunit (*green*). *Note* unique ER perinuclear staining pattern for FSHβ-L mutant (B, *arrow*) vs. dispersed cytoplasmic puncta for LHβΔT and LHβL119A subunit (A, C, *arrowhead*) or LH dimer (D, *arrowhead*). The n indicates the nucleus (*red*). The micrographs shown are representative of four to eight experiments. IgG (E), mouse immunoglobulin. ×100.

Because the heptapeptide is critical for LH sorting, we suspected that NE localization of LHβ was due to this sequence. To test this prediction, we stained cells expressing LHβΔT; no distinctive perinuclear staining was observed (Fig. 4A). To further examine the role of LHβ heptapeptide, GH_3_ cells expressing a chimera comprised of the FSHβ gene fused to the sequence encoding the heptapeptide (FSHβ**-**L**)** were immunostained with a monoclonal antibody against the FSHβ subunit (Fig. 4B). If the perinuclear staining of the LHβ subunit is attributed to the heptapeptide, the FSHβ-L chimera should also exhibit a comparable staining pattern. Similar to LHβ, the FSHβ-L chimera displayed a perinuclear-staining (67.9±2.6% of cells; n >200 cells; Fig. 4B; Table 1). As expected, mouse IgG exhibited no detectable staining (Fig. 4E).

**Table 1:** Summary of subunit/chaperone localization in the ER of GH_3_ cells.

		ER Localization	
Subunit/Chaperone	Heterodimer Secretion	Perinuclear	Peripheral
LHβ	Regulated	+	+
LHβΔT	Constitutive	−	+
LHβL119A	Constitutive	−	+
FSHβ	Constitutive	−	+
FSHβ-L	Regulated	+	+
BiP	NA	+	+
CNX	NA	+/−	+/−

Previously we identified a dileucine motif in the heptapeptide that accounted for directing LH dimer to the regulated pathway [24]. This predicts that mutating the determinant Leucine 119 to Alanine in the LHβ subunit (LHβL119A, Fig. 1) should reduce the staining of the mutant in the NE region. The LHβL119A mutant showed uniform cytoplasmic staining (Fig. 4C) rather than accumulation in the NE region characteristic for the LHβ subunit. The next experiments addressed the question of whether the LH heterodimer is also targeted to the NE. GH_3_ cells expressing LH dimer, and immunostained with CGβ polyclonal antiserum, exhibited no distinct localization in the NE region (Fig. 4D). Thus, the accumulated LHβ subunit is displaced from the NE region of the ER to peripheral ER upon combination with the α subunit. The results confirm that only β subunits bearing the heptapeptide accumulate in the perinuclear region and this sequence is responsible for targeting the non-assembled LHβ subunit to this area.

**Figure 5:**
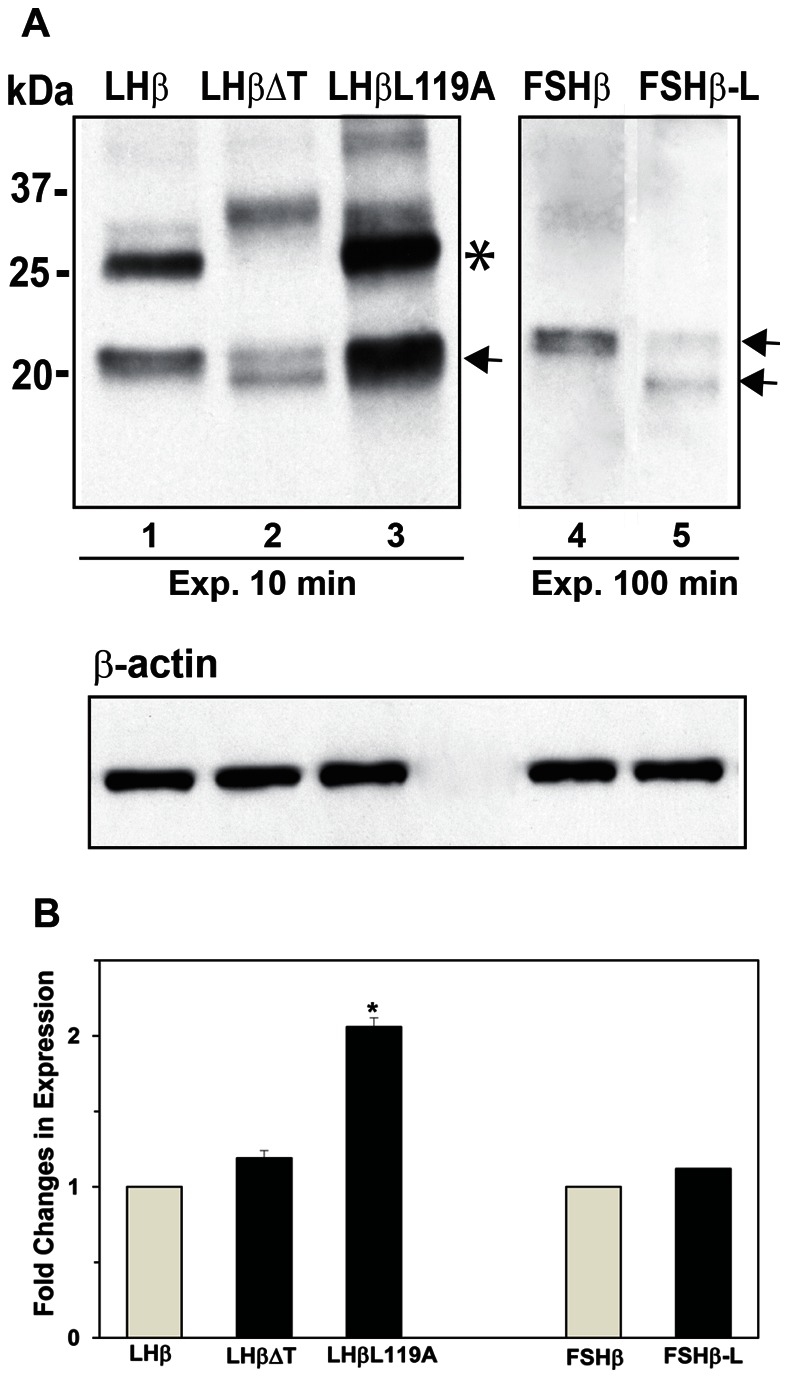
Representative Western blot of cell lysates (50 µg total protein/lane) derived from GH_3_ cells. (**A**) The migration of subunits (*arrows*) and molecular mass markers are indicated. *Note* the longer time exposure (Exp.) for FSHβ and FSHβ-L (lanes 4 and 5) compared to LHβ and mutants (lanes 1–3). Bands at approximate 25 kDa presumably represents protein aggregates (*). In addition, LHβΔT and FSHβ-L are separated on SDS-PAGE gel into 2 bands (*arrows*). β-Actin was used as an internal control. (B). Histogram of densitometric measurements for LHβ, FSHβ and mutants. The protein level for LHβ and FSHβ was arbitrarily set as 1. Fold changes in expression level of LHβ mutants and FSHβ-L were compared with LHβ and FSHβ, respectively. Each value indicates the mean **±** SEM (n = 3). *Significant difference from LHβ with *p*<0.05.

To examine if the different staining patterns for LHβ, FSHβ and mutants were influenced by their intracellular expression levels, lysates of the GH_3_ lines synthesizing individual subunits were examined by Western blotting (Fig. 5). LHβ and its variants migrated at 20–22 kDa (Fig. 5A, lanes 1–3; arrow). The expression of LHβΔT and LHβL119A was 1.2 and 2-fold higher, respectively, compared to the level of LHβ (Fig. 5B). It is unclear as to the identity of the proteins migrating at approximately 25 kDa (Fig. 5A, asterisk), but it is likely due to aggregation and because they are not observed under reduced conditions as previously shown [25]. Thus, it is evident that the lack of staining in the perinuclear region for LHβΔT and LHβL119A are not due to their reduced synthesis (Fig. 5A, lanes 2, 3) compared to LHβ (Fig. 5A, lane 1). FSHβ and FSHβ-L (detected as 2 bands) show comparable protein levels (Fig. 5A, lanes 4, 5, 5B). To detect the FSHβ and FSHβ-L subunits, it was necessary to expose blots 10-fold longer time than for the LHβ (Fig. 5A). This difference in sensitivity may be related to variations in antibody affinities. While we cannot exclude expression of LHβ (and its analogs) are more robust, that the sensitivities for FSHβ and FSHβ-L are similar implies that the immunoreactivity of the FSHβ antibody is less than the corresponding LHβ immunoprobe. Since the protein levels of FSHβ and FSHβ-L are comparable – but only the mutant displays significant perinuclear staining – the lack of perinuclear FSHβ staining is not related to differential intracellular expression levels, but rather the presence of the heptapeptide sequence in the FSHβ-L chimera.

**Figure 6:**
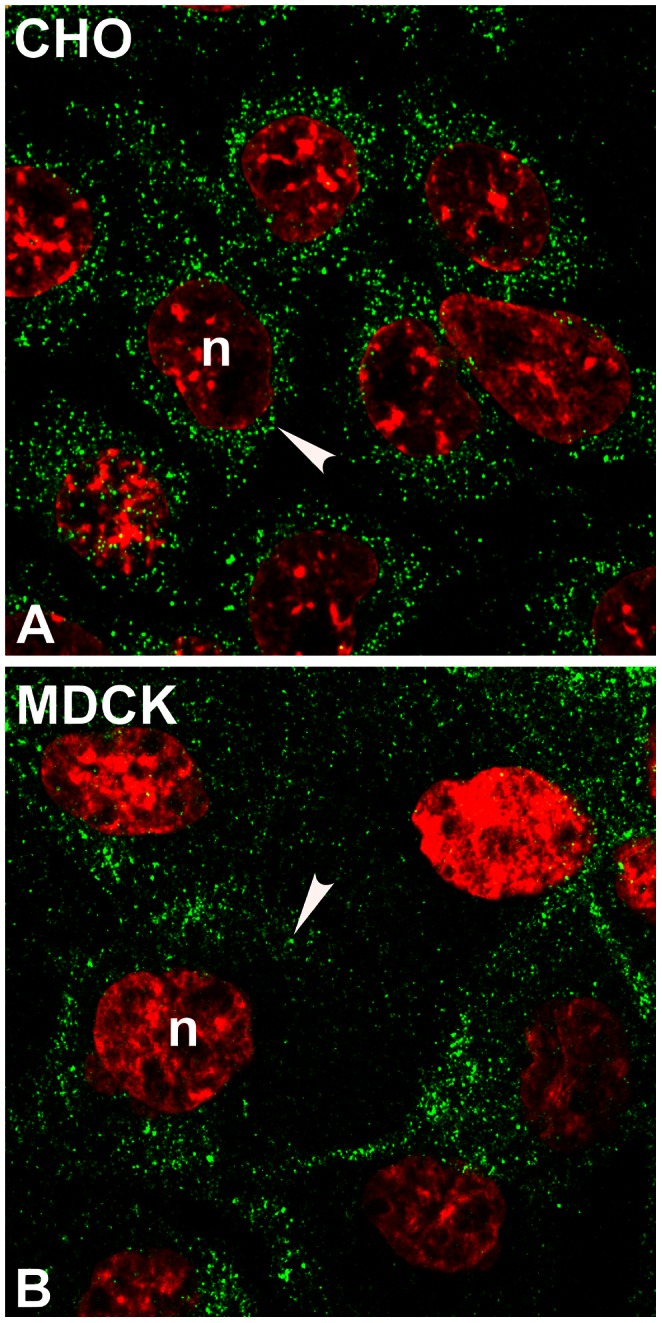
Immunostaining of LHβ subunit in CHO (A) and MDCK (B) cells. The cells were immunoprobed with CGβ antiserum (*green*). *Note* that LHβ shows dispersed cytoplasmic puncta (A, B, *arrowhead*) with no ring-like pattern near nucleus. The n indicates the nucleus (*red*). The micrographs shown are representative of four experiments. X150.

Because CHO and MDCK cells lack a regulated secretory pathway, we also examined the fluorescence staining of the LHβ subunit in these cells (Fig. 6). In contrast to GH_3_ cells, both cell lines expressing LHβ showed only dispersed cytoplasmic puncta with no detectable perinuclear staining (Fig. 6A, B). The data imply that the LHβ staining in the NE region of GH_3_ cells is associated with cells secreting protein via the regulated route.

**Figure 7:**
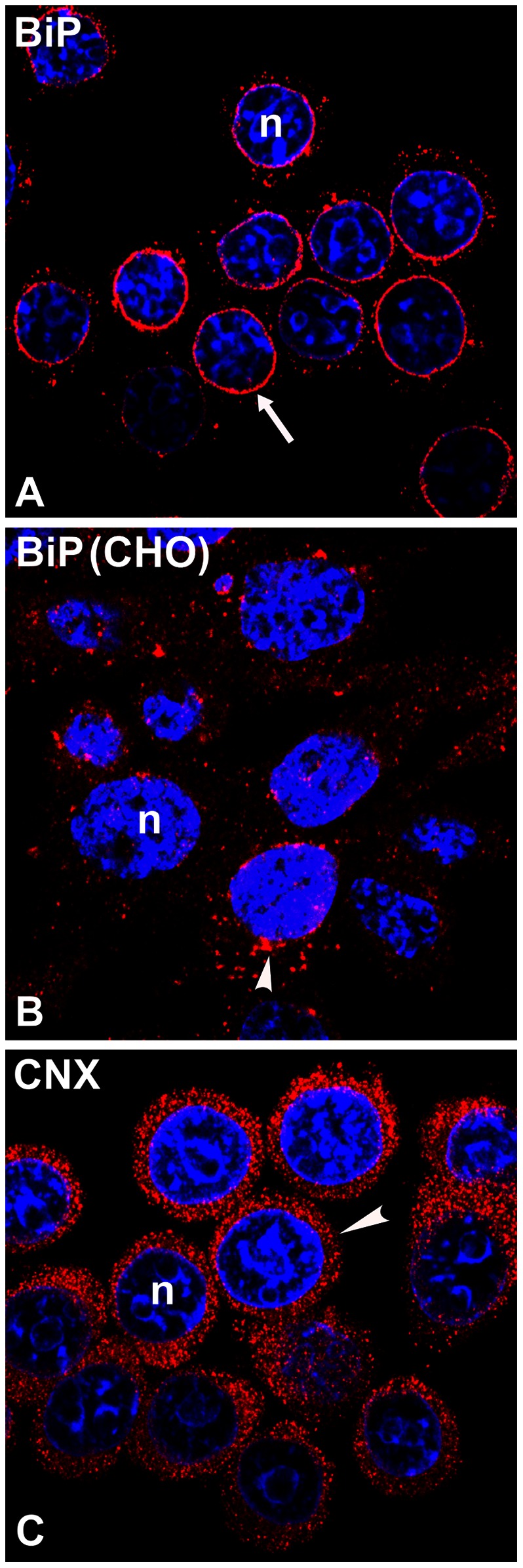
Immunolocalization of endogenous BiP (A, B) and calnexin (CNX, C) in non-transfected GH_3_ or CHO cells. For GH_3_ cells the BiP antiserum (A, *red*) stained predominantly around nuclei (*arrow*), while the CNX antiserum (C, *red*) showed peripheral ER staining (*arrowhead). Note* that BiP in CHO cells (B) is localized as dispersed cytoplasmic puncta with some aggregation near the NE (*arrowhead*). Nuclei (n) were counterstained using TOPRO-iodide-3 (*blue*). The micrographs shown are representative of four experiments.

The preferential staining of LHβ in the ER region of the nuclear envelope in GH_3_ cells compared to peripheral ER staining suggests that the spatial separation might coincide with selective chaperone binding. To address this point, we examined the localization of two endogenous ER chaperones (Fig. 7), immunological heavy chain-binding protein (BiP) and calnexin (CNX). BiP is localized to the ER lumen [26], [27], and CNX is an integral ER membrane protein and both contribute to early protein folding events in the secretory pathway [28]–[30]. Single staining of non-transfected GH_3_ cells with BiP antiserum revealed an intense signal predominantly located in the perinuclear area forming a punctate ring with some staining in the cell periphery (Fig. 7A, Table 1), which has also been shown by others [31]. In contrast, CNX exhibited generalized ER staining throughout the cell (Fig. 7C). The implication of these data is that the prominence of BiP staining in the perinuclear region of the ER might be related to the presence of the regulated pathway in GH_3_ cells. To address this point, we examined staining pattern of endogenous BiP in CHO cells, which secrete proteins primarily through the constitutive pathway. In contrast to GH_3_ cells, BiP staining in CHO cells is not concentrated to the nuclear envelope, but rather scattered throughout the cell (Fig. 7B). These data imply that the prominent nuclear envelope/ER staining of BiP in GH_3_ cells is associated with the regulated secretion pathway.

**Figure 8:**
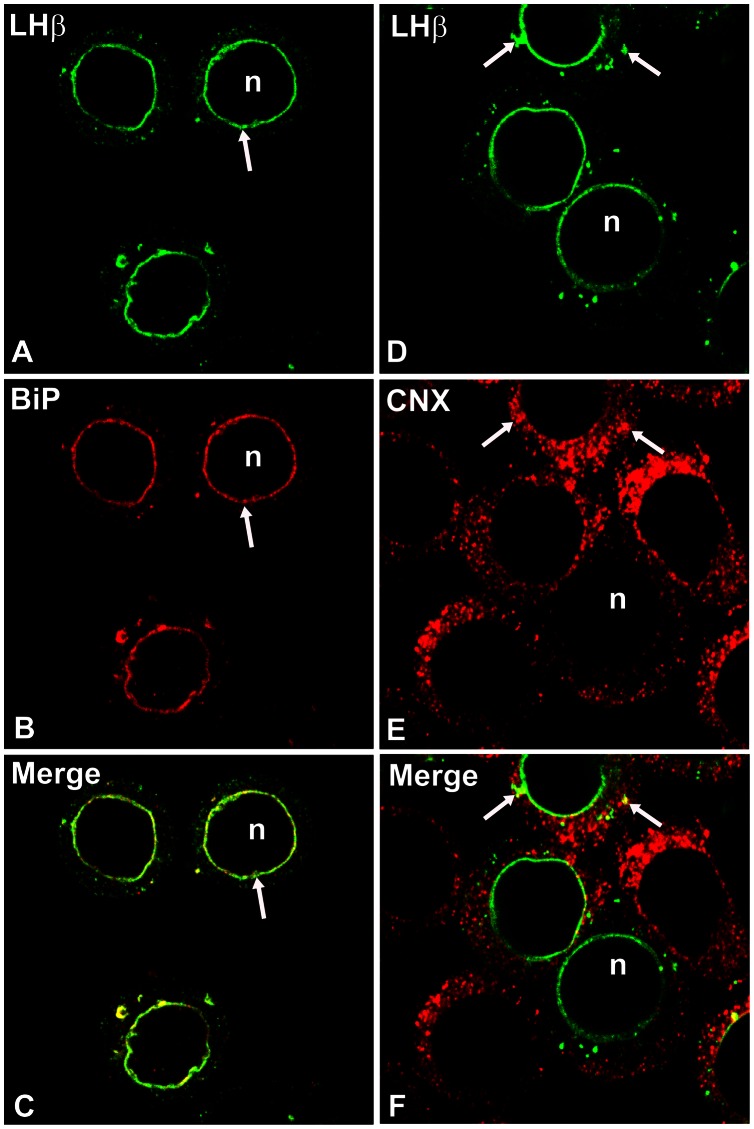
Dual immunostaining of LHβ expressing cells with endogenous BiP (A–C) or calnexin (CNX, D–F). GH_3_ cells were immunostained with LHβ monoclonal antibody (*green*) and BiP (*red*) or CNX (*red*) antisera. The yellow color in merged images indicate co-localization (C, F). *Note* that both LHβ and BiP display ring-like patterns near nuclei (*arrows*). There is a significant co-localization of LHβ subunit with BiP (Pearson’s correlation coefficient, r = 0.832**±**0.014, *p*<0.01). In contrast, LHβ subunit shows a weak co-staining with CNX (Pearson’s correlation coefficient, r = 0.252**±**0.021). These images are representative of four independent experiments. X150.

To examine the LHβ subunit co-localization with ER chaperones, dual stainings were performed with a monoclonal antibody against LHβ, and polyclonal antisera against BiP or CNX (Fig. 8). Significant co-localization of LHβ and BiP in the perinuclear region (Pearson’s correlation coefficient, r = 0.832±0.014, *p*<0.01) indicated by yellow color in the merged image (Fig. 8C) implies the unique ER retention of unassembled LHβ is co-incident with BiP in the same ER sub-domain. In contrast, only some co-staining of LHβ with CNX was detected (Pearson’s correlation coefficient, r = 0.252±0.021) in the NE and in the peripheral regions of the ER (Fig. 8, arrow). These data suggest that the presence of BiP drives the accumulation of LHβ in the NE region.

## Discussion

Our prior findings indicated that the C-terminal heptapeptide in the LHβ subunit was associated with a complex of intracellular determinative actions regarding the secretory fate of LH dimer: Extent of assembly [10], [32], basolateral release from the pituitary [33], and controlling entry into the regulated pathway [16]. Here, we identified another feature of the heptapeptide, its ability to direct the LHβ subunit to a perinuclear sub-domain of the ER, which is distinct from localization of the FSHβ subunit. Our conclusion is based on: 1) localization of the LHβ subunit to the perinuclear region of cells, 2) no detectable perinuclear staining of the LHβΔT and LHβL119A mutants, and 3) dispersion of FSHβ subunit fluorescence throughout the peripheral ER, with perinuclear staining for the FSHβ-L chimera. These data support a model in which the regulated biosynthetic routing of LH is initiated at a sub-domain of the ER, the nuclear envelope region, and depends on the presence of the LHβ heptapeptide sequence.

We further validated our conclusion by examining LHβ localization in transfected CHO and MDCK cells, which secrete proteins only constitutively [18], [33]. This additional set of experiments permitted us to ask whether the LHβ perinuclear-staining pattern is unique to cells containing the regulated pathway. No significant perinuclear staining was observed in either CHO or MDCK cells, rather, only dispersed cytoplasmic puncta were detected, indicative of peripheral ER localization. In contrast to the single LHβ subunit data, no significant perinuclear staining of the assembled LH dimer was evident in GH_3_ cells. Essentially all of the fluorescence was observed as dispersed puncta in areas of the peripheral ER. The ability of heterodimer formation to successfully release the LHβ or FSHβ-L pool from the ER/nuclear envelope region is in agreement with our previous claim [9] that the α subunit serves as an escort/chaperone to further traffic the LH heterodimer through the regulated secretory pathway.

Studies in other systems have shown that proteins can interchange between the peripheral ER domains/NE [34], [35]. For example, TorsinA (TorA), a member of the AAA+ ATPase family, is an ER protein required for normal neurological function. Although TorA resides in the peripheral ER, its primary site of action is at the nuclear envelope. The distribution of TorA in the ER/NE is related to the levels of endogenous ER transmembrane proteins and variations in the expression of these proteins results in redistribution of TorA in the ER/NE. In addition, site-directed mutagenesis of a hydrophobic amino terminal stretch in TorA also alters the distribution between ER/NE.

Several recent reports describe the ER as a mosaic of specialized sub-domains, which have distinct functions, as well as a specific distribution of resident proteins [36]–[43]. Moreover, the ER-resident membrane Sec61 complex that comprises the translocon is present in the nuclear envelope [44]. These data support the hypothesis that the transfer of LH during its biosynthetic maturation involves more than one ER compartment, and implicate BiP in this schema. BiP facilitates the proper folding and assembly of multi-subunit complexes and it associates with the incompletely folded human CGβ subunit - which shares 85% amino acid identity with the LHβ subunit - resulting in a mature assembly-competent subunit [45], [46]. Moreover, the primary interactions between BiP and polypeptides occur at small hydrophobic patches of 7–9 amino acids [47], [48]. Thus, we suggest that BiP occupies the heterodimer interface of the LHβ subunit and is subsequently displaced by the α subunit resulting in movement of LH dimer from the perinuclear to the peripheral region of the ER and exits to the cis Golgi. The co-localization of LHβ and BiP at perinuclear sites supports this conclusion. LH may also enter the secretory pathway in vesicles that bud directly from the NE. It has been demonstrated that the COP II and, to a lesser extent COP I vesicles, are known to bud from the NE [49]–[51].

In summary the data imply that both the ER and trans-Golgi are critical for gonadotropin sorting. The first sub-domain segregation of LH and FSH synthesis occurs in the ER and subsequently, protein transfer to the Golgi leads to recognition of sorting motifs in the hormone and packaging to unique vesicle populations. This model provides an explanation of how an intracellular pool of non-combined α, LHβ and FSHβ subunits can assemble in the ER to generate LH and FSH heterodimers, and ultimately sorting them to their distinct regulated and constitutive secretion pathways.

## Materials and Methods

### Reagents and Antibodies

Ham’s F-12 medium, DMEM/F12, Dulbecco’s phosphate-buffered saline (DPBS), L-glutamine, trypsin and penicillin/streptomycin were obtained from Fisher Scientific (Pittsburgh, PA). The neomycin analog G418 was obtained from Research Product International (Mt. Prospect, IL). Normal rabbit serum and bovine serum albumin (BSA) were purchased from Sigma (St. Louis, MO). Fetal bovine serum (FBS) and horse serum (HS) were obtained from Harlan Bioproducts for Science, Inc. (Indianapolis, IN) and Gibco (Grand Island, NY), respectively. [^35^S]Cysteine was obtained from MP Biomedicals, Inc. (Irvine, CA). Lipofectamine 2000 and Pansorbin were purchased from Invitrogen Corp. (Carlsbad, CA) and EMD BioSciences Inc. (La Jolla, CA), respectively. Normal goat serum, mouse IgG and VectaShield mounting medium were purchased from Vector Laboratories (Burlingame, CA). Antiserum against α or CGβ (which also detects LHβ but does not cross react with the α subunit) subunits were prepared in our laboratory. Monoclonal antibody against nuclear pore complexes (mAb414) was purchased from Covance (Princeton, NJ). Antiserum against BiP was a gift from Linda Hendershot (St. Jude Children’s Research Hospital, Memphis, TN) [26], [27] and CNX antiserum was purchased from Enzo Life Sciences (Plymouth Meeting, PA). The β**-**actin monoclonal antibody was purchased from Sigma (St. Louis). Monoclonal antibodies against human LHβ and FSHβ subunits were a gift from Organon (B.V.) [17], [24]. TOPRO-iodide-3, goat anti-mouse IgG and goat anti-rabbit IgG conjugated to Alexa Fluor 488 or conjugated to Alexa Fluor 568 were bought from Invitrogen Corp. (Carlsbad). Protein Assay was obtained from Bio-Rad Laboratories (Hercules, CA). Tropix Chemiluminescent Substrate, Tropix Nitro-Block Luminescence Enhancer, I-Block, goat anti-mouse IgG and goat-anti rabbit IgG conjugated to alkaline phosphatase were purchased from Applied Biosystems (Foster City, CA). Complete protease inhibitor cocktail tablets were from Roche Diagnostic (Indianapolis, IN).

### Cell Culture, Transfection and Selection of Stable Cell Clones

GH_3_ cells were a gift from the late Dr. Dennis Shields (Albert Einstein College of Medicine, New York, NY) [16]–[18]. The cells were grown (no more than 35 passages) at 37°C in Ham’s F-12 medium supplemented with 12.5% HS, 2.5% FBS, 2 mM L-glutamine, 100 U/ml penicillin, and 100 µg/ml streptomycin in a humidified 5% CO_2_ incubator. CHO (from American Type Culture Collection) [8]–[10] and MDCK (strain II, gift of Dr. Sharon Milgram from University of North Carolina, Chapel Hill, NC) [33] cells were cultured in Ham’s F12 or DMEM/F12, respectively, supplemented with 5% FBS, 2 mM L-glutamine, 100 U/ml penicillin, and 100 µg/ml streptomycin. Cells were transfected with genes encoding α, LHβ, LHβ114 (designated LHβΔT), LHβL119A, FSHβ or FSHβ chimera (designated FSHβ-L) subunits (Fig. 1) using vector pM^2^ HA [16]. The mutant LHβΔT described previously [8] lacks a seven-amino acid extension (Leu-Ser-Gly-Leu-Leu-Phe-Leu) at the C terminus of the LHβ subunit. The mutant LHβL119A was constructed (Fig. 1) where Leucine119 codon was mutated to Alanine [24]. To construct the FSHβ-L chimera, the heptapeptide sequence of the LHβ subunit (plus a stop codon) was inserted in-frame at the 3′-end of the FSHβ subunit [16]. Transfection was performed using Lipofectamine 2000 on semi-confluent cells in 6-well plates according to the manufacturer’s instructions [16], [17], [24]. Stable clones were selected with 0.25 mg/mL of G418. Single colonies were isolated and subsequently screened by immunoprecipitating proteins from the media and lysates of [^35^S] cysteine labeled cells. Several clones (n = 5 per subunit) were maintained in culture and used for the experiments described below.

### Immunofluorescence and Confocal Microscopy

Single or double-stained immunofluorescence microscopy was performed to assess the subcellular distribution of the (A) glycoprotein subunits, (B) nuclear pore complexes (the NE marker, mA414), and (C) the ER chaperones, BiP and CNX. GH_3_, CHO, and MDCK cells expressing subunits were grown on Fisherbrand Superfrost-Plus microscopy slides (Fisher Scientific, Pittsburg) in Petri dishes. The cells were fixed with 4% paraformaldehyde for 20 min at room temperature (RT) and permeabilized with 0.2% Tween-20 (diluted in DPBS) for 10 min [16]. Cells were then incubated in 20% normal goat serum for 1 h to block nonspecific binding and washed three times for 10 min in 2% BSA in DPBS. Cells were incubated at RT with primary antibodies (1∶250–1∶1000 dilution in 2% BSA/DPBS) for 30–60 min, washed and stained with goat anti-rabbit IgG conjugated to either Alexa Fluor 488 or to Alexa 568 (1∶250 dilution) and goat anti-mouse IgG conjugated to Alexa Fluor 488 or conjugated to Alexa 568 for 20 min. Following three washes in 2% BSA/DPBS, and once in DPBS, nuclei were counterstained with TOPRO-iodide-3 (1∶500 diluted in DPBS) for 15 min. After several washes with DPBS, the cells were mounted in VectaShield mounting medium. Negative controls for polyclonal antisera or monoclonal antibodies were normal rabbit serum or mouse lgG, respectively. Immunostaining against nuclear pore complex proteins was performed at 4°C.

To determine whether LHβ subunit co-localizes with endogenous ER chaperones, GH_3_ cells were double immunostained with LHβ monoclonal antibody, plus BiP or CNX polyclonal antiserum followed by incubation with Alexa Fluor 488 (green fluorescence for LHβ) and Alexa Fluor 568 (red fluorescence for BiP and CNX)-conjugated secondary antibodies. Control immunostaining of cells incubated either with two primary antibodies and one secondary antibody, or with one primary and two secondary antibodies were also performed. The corresponding single staining for LHβ, BiP or CNX was also included in these experiments.

Confocal imaging was performed with an Olympus FV-500 confocal microscope with a z-interval of 0.5 µm using x100 oil objective (image size 1024×1024 and 512×512 pixel images). All confocal images represent the sum of 4–6 adjacent confocal planes from the stack and a zoom setting of 1 and 1.5. For dual staining, green and red immunofluorescence was imaged sequentially to ensure no overlapping excitation between channels. Processing of images was performed using the Metamorph Image software package (Molecular Devices Corp., Downington, PA). Maximum intensity projections of confocal z-series were made in Image J (v1.4, NIH, Bethesda, MD). Images were assembled in Adobe Photoshop (CS3) and panels were labeled in Adobe Illustrator (CS3).

### Western Blot Analysis

Intracellular expression of LHβ, LHβΔT, LHβL119A, FSHβ, and FSHβ-L proteins were examined in lysates by Western blotting. After termination of culture, cells were washed with ice-cold DPBS and lysated in the presence of protease inhibitor cocktail. After centrifugation protein concentrations in supernatants were determined with the Bradford reagent using BSA as a standard. For LHβ, FSHβ and mutants, 50 µg of proteins were resolved on 15% SDS-PAGE in the absence of heat or reducing agent and transferred onto nitrocellulose. The LHβ or FSHβ monoclonal antibodies were incubated for 1 hour at RT in DPBS with 0.1% Tween-20. The β-actin monoclonal antibody was used as an internal control. The membranes were probed with alkaline phosphatase-coupled secondary antibodies for 1 hour at RT and developed using Tropix chemiluminescence substrate.

### Analysis of Data

For each gonadotropin subunit and dimer, the percentage of cells showing the perinuclear staining pattern was calculated in 5–8 fields per slide (200–700 cells). That only LHβ and FSHβ-L showed the perinuclear pattern, their data (mean ± SEM; n = 5 experiments) were analyzed by t-test, with *p*<0.05. The bands from Western blots were densitometrically scanned using a GS-710 calibrated Imaging Densitometer and quantified using the Quantity One Software (BioRad Laboratories Inc.). The protein level for LHβ and FSHβ was arbitrarily set as 1 and fold changes in the expression level of LHβ mutants and FSHβ-L were compared with LHβ and FSHβ, respectively. Statistical analysis was performed by *t*-test. Each experiment was repeated four-eight times and the results are expressed as mean ± SEM, with *p*<0.05 considered significantly different. Co-localization between LHβ and BiP or CNX was calculated with an ImageJ using JACoP program [52], [53] and expressed as Pearson’s correlation coefficient (rCC). The RCB images were converted to an 8-bit grayscale and an automatically detected threshold was applied to eliminate the background. The rCC, which can range from −1 to +1, greater than 0.69 was considered to indicate significant co-localization [52], [53]. Co-localization was analyzed in 5–10 fields in a single experiment. Each experiment was repeated three to five times and the results are expressed as mean ± SEM. Statistical significance was performed by *t*-test with *p*<0.01.
